# The Interplay Between Programmed Death Ligand 1 and Vimentin in Advanced Non-Small-Cell Lung Cancer

**DOI:** 10.3389/fonc.2021.669839

**Published:** 2021-05-04

**Authors:** Giuseppe Bronte, Maurizio Puccetti, Elisabetta Petracci, Lorenza Landi, Paola Cravero, Simona Scodes, Paola Ulivi, Sara Ravaioli, Maria Maddalena Tumedei, Marco Angelo Burgio, Federico Cappuzzo, Angelo Delmonte, Lucio Crinò, Sara Bravaccini

**Affiliations:** ^1^ Department of Medical Oncology, IRCCS Istituto Romagnolo per lo Studio dei Tumori (IRST) “Dino Amadori”, Meldola, Italy; ^2^ Pathology Unit, Azienda Unitá Sanitaria Locale (AUSL) Imola, Imola, Italy; ^3^ Unit of Biostatistics and Clinical Trials, IRCCS Istituto Romagnolo per lo Studio dei Tumori (IRST) “Dino Amadori”, Meldola, Italy; ^4^ AUSL Romagna, Ospedale Santa Maria delle Croci, Ravenna, Italy; ^5^ Biosciences Laboratory, IRCCS Istituto Romagnolo per lo Studio dei Tumori (IRST) “Dino Amadori”, Meldola, Italy

**Keywords:** vimentin, programmed death ligand 1, non-small-cell lung cancer, epithelial-to-mesenchymal transition, immunohistochemistry

## Abstract

**Background:**

Current therapy for non-small-cell lung cancer (NSCLC) frequently includes immune checkpoint inhibitors, such as pembrolizumab, and programmed death ligand 1 (PD-L1) positivity is mandatory for its use in this setting. Vimentin plays a role in carcinogenesis through the activation of the epithelial-to-mesenchymal transition (EMT) process. Its prognostic impact in NSCLC has been investigated in numerous studies but little data are available on its relation with PD-L1 expression.

**Patients and Methods:**

We retrospectively retrieved data on patients with advanced NSCLC consecutively enrolled in a clinical trial at our institute. PD-L1 and vimentin expression were determined by immunohistochemistry. Correlations between variables were assessed using the Spearman correlation coefficient. The Kaplan-Meier method was used to estimate overall survival (OS) and the Log-rank test was used to compare survival curves. The association between demographic, clinical and biomarker information and survival was investigated with the Cox model.

**Results:**

Fifty-three patients were included in the study. A weak positive correlation was observed between the PD-L1 and vimentin (ρ=0.41, *P*=0.003). Patients with PD-L1 values <1% showed a slightly better OS than those with higher values (HR=2.07; 95% CI: 0.92–4.65), but the difference was not significant (*P*=0.080). No difference in overall survival (OS) was observed on the basis of vimentin expression (HR=1.25; 95% CI: 0.59–2.66; *P*=0.554). Patients harboring both vimentin and PD-L1 negative expression (<1%) showed a trend towards better survival than those with ≥1% expression (HR=2.31; 95% CI: 0.87-6.17, *P*=0.093). No significant associations were observed between gender, age at diagnosis, stage at diagnosis, histology, KRAS or EGFR status, radical surgery or immunotherapy and OS.

**Conclusions:**

The weak positive association between PD-L1 and vimentin suggests a potential interplay between these biomarkers. Further research is warranted to evaluate EMT and immune escape as two components of the same process.

## Introduction

The therapeutic strategy for non-small cell lung cancer (NSCLC) has radically changed since the advent of immune checkpoint inhibitors. The majority of these agents target programmed death 1 (PD-1) and its ligand (PD-L1). The PD1/PD-L1 axis suppresses the antitumor immune response by exploiting the mechanisms of immune tolerance. PD-L1, a cell surface protein physiologically expressed in numerous tissues, binds PD-1 expressed on the surface of cytotoxic T cells, causing anergy or apoptosis (1). Similarly, many tumors express PD-L1 as a mechanism of immune escape (2).

Currently PD-L1 expression is only mandatory for the prescription of pembrolizumab, which is indicated as first-line treatment if ≥ 50% of tumor cells are positive for PD-L1, and as second-line therapy if PD-L1 expression is found in >1% tumor cells (3, 4). Conversely, nivolumab and atezolizumab, the other immune checkpoint inhibitors approved for NSCLC, do not require positive tumor PD-L1 expression to be used (5–8).

PD-L1 as a predictive biomarker has several limitations such as the need for 4 different antibodies to assess it, and the heterogeneity of its expression within the same tumor and during tumor evolution and treatments (9, 10). For these reasons the biological mechanisms regulating its expression and function require further investigation.

Vimentin, a type III intermediate filament protein, is constitutively expressed in some mesenchymal cells (*e.g.* macrophages, fibroblasts, endothelial cells). During cancer progression, its expression is regulated by transcriptional regulation. Vimentin is also expressed by epithelial-derived tumor cells, with expression higher in metastatic cancer cells than primary tumors (11). Thus, vimentin plays a role in carcinogenesis through the activation of the epithelial-to-mesenchymal transition (EMT) process. However, it can also influence sensitivity to epidermal growth factor receptor (EGFR)-directed tyrosine kinase inhibitors (TKIs).When cancer cells undergo EMT they usually acquire stem‐like properties, leading to epithelial cancer dissemination (12, 13). EMT‐related and stem cell‐related transcription factors are induced by paracrine and autocrine stromal factors (*e.g.* TGF‐β). Consequently, cancer stem cells (CSCs) are formed through the acquisition of mesenchymal markers (14, 15).

This process is characterized by epigenetic modifications in histones and in DNA, resulting in a progressive modification of the expression of specific genes encoding for transcription factors and mesenchymal markers. These epigenetic changes have different degrees of stability for the maintenance of the mesenchymal phenotype. Thus, we can consider the EMT process as an adaptation to the microenvironment (16). Some *in vitro* studies have suggested that the TGF‐βR pathway is predominant in triggering EMT (17).

Numerous studies have explored the prognostic role of vimentin expression. An important meta-analysis by Ye et al. summarized findings from 32 studies including 4118 patients on this topic. The pooled hazard ratio (HR) for OS indicated that vimentin overexpression in NSCLC was significantly associated with a worse prognosis, highlighted in univariate analysis but not in multivariate analysis (18).

In the present paper we explore whether the expression of PD-L1 and vimentin are associated and whether this association has a prognostic impact.

## Patients and Methods

### Study Design

This was a retrospective cohort study designed to explore the correlation between vimentin and PD-L1 expression and their separate and combined effect with respect to OS. We hypothesized that vimentin positivity is associated with PD-L1 positivity and that there is a positive correlation between the two biomarkers. We also conjectured that patients with both vimentin and PD-L1 negative immunohistochemical (IHC) expression have a better prognosis in terms of OS than those with one negative biomarker or both positive biomarkers.

### Patients

For the present retrospective work we retrieved data on patients with NSCLC consecutively enrolled at Istituto Scientifico Romagnolo per lo Studio e la Cura dei Tumori (IRST) IRCCS and Area Vasta Romagna (AVR) after January 2017. Eligibility criteria were age ≥18 years, histological diagnosis of NSCLC and radiological evidence of advanced disease. At least one specimen from primary tumor or metastasis had to be available. Consulting the clinical records, we extracted information on histology, stage at diagnosis, date of diagnosis, tumor mutation status with regard to KRAS, EGFR, ALK and ROS1, radical surgery, lines of treatment for advanced disease, and date of death or date of last follow-up visit.

The study was reviewed and approved by the Ethics Committee of IRST and Area Vasta Romagna (code: L3P1490; approval no. CE 1240/2017) and was carried out in accordance with the principles laid down in the Declaration of Helsinki. Written informed consent was obtained from patients.

### Immunohistochemistry

**Figure 1:**
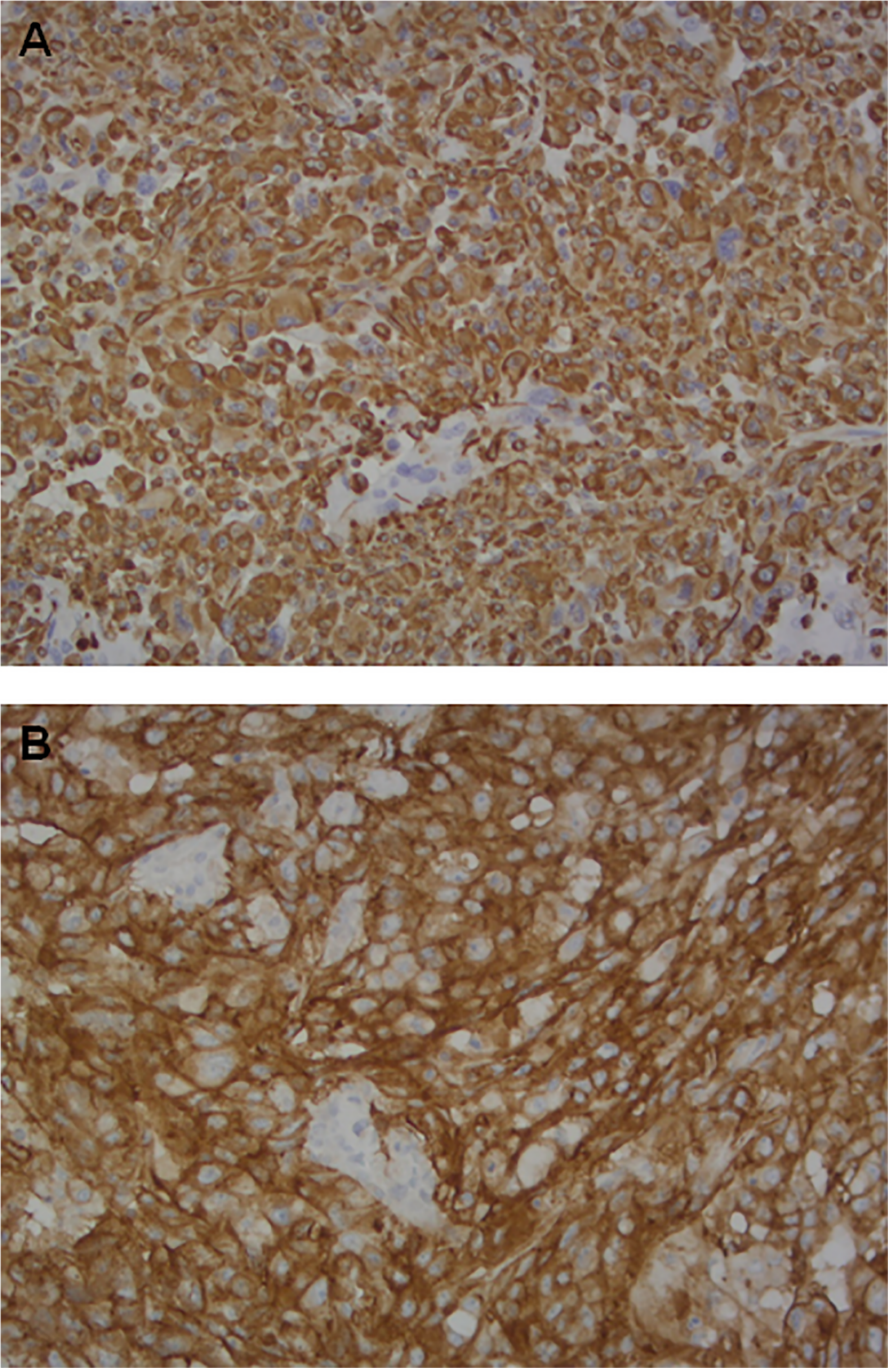
Paraffin-embedded stained section of tissue samples showing similarly strongly positive epithelial cells from a patient with squamous cell carcinoma (SCC) of the lung stained on the Ventana BenchMark XT platform using: **(A)** SP263 Roche-Ventana antibody: PD-L1 (magnification×20); **(B)** Confirm anti-Vimentin V9 antibody: vimentin (magnification ×20).

Tumor material obtained during surgery was fixed in neutral buffered formalin and embedded in paraffin. Four-micron sections were mounted on positive-charged slides for each patient (Bio Optica, Milan, Italy). Biomarker determinations were performed according to European Quality Assurance guidelines. Immunostaining for PD-L1 and vimentin expression was carried out using the Ventana Benchmark XT staining system (Ventana Medical Systems, Tucson, AZ, USA) with the Optiview DAB Detection Kit (Ventana Medical Systems) (**Figure 1**).

PD-L1 SP263 and Confirm anti-Vimentin V9 (Ventana Medical Systems) antibody clones were used. For their detection, tissue sections were incubated for 16 minutes with prediluted antibodies according to the manufacturer’s recommendations. Sections were then incubated for 16 minutes and automatically counterstained with hematoxylin II (Ventana Medical Systems). Placenta was used as positive control for both biomarkers. Membrane biomarker positivity was detected and semiquantitatively quantified as the percentage ratio between immunopositive tumor cells and the total number of tumor cells. All samples were evaluated by 2 independent observers and any disagreement (>10% positive cells for the different markers) was resolved by consensus after joint review using a multihead microscope.

### Statistical Analysis

Data were summarized using mean ± standard deviation (SD) or median and minimum and maximum values, as appropriate, for continuous variables. Categorical variables were reported as natural frequency and percentage. Correlation among variables was measured using the Spearman correlation coefficient. Given the singular ‘spike at zero’ distribution of the two biomarkers considered in this study, the association with OS was assessed using dichotomized variables for a 1% value. Patients with expression values < 1% were considered negative for the biomarkers, while with values ≥ 1% were considered positive. OS was defined as the time from the start of first-line treatment for advanced disease until death from any cause or last patient visit by January 2019. The Kaplan-Meier method was used to estimate the OS function and the Log-rank test was used for survival curve comparison. The association between demographic, clinical or biomarker information and survival was investigated with the Cox model. The proportional hazards assumption was tested using Schoenfeld residuals. Results were reported as hazard ratios (HRs) and 95% confidence intervals (CIs).

## Results

**Figure 2:**
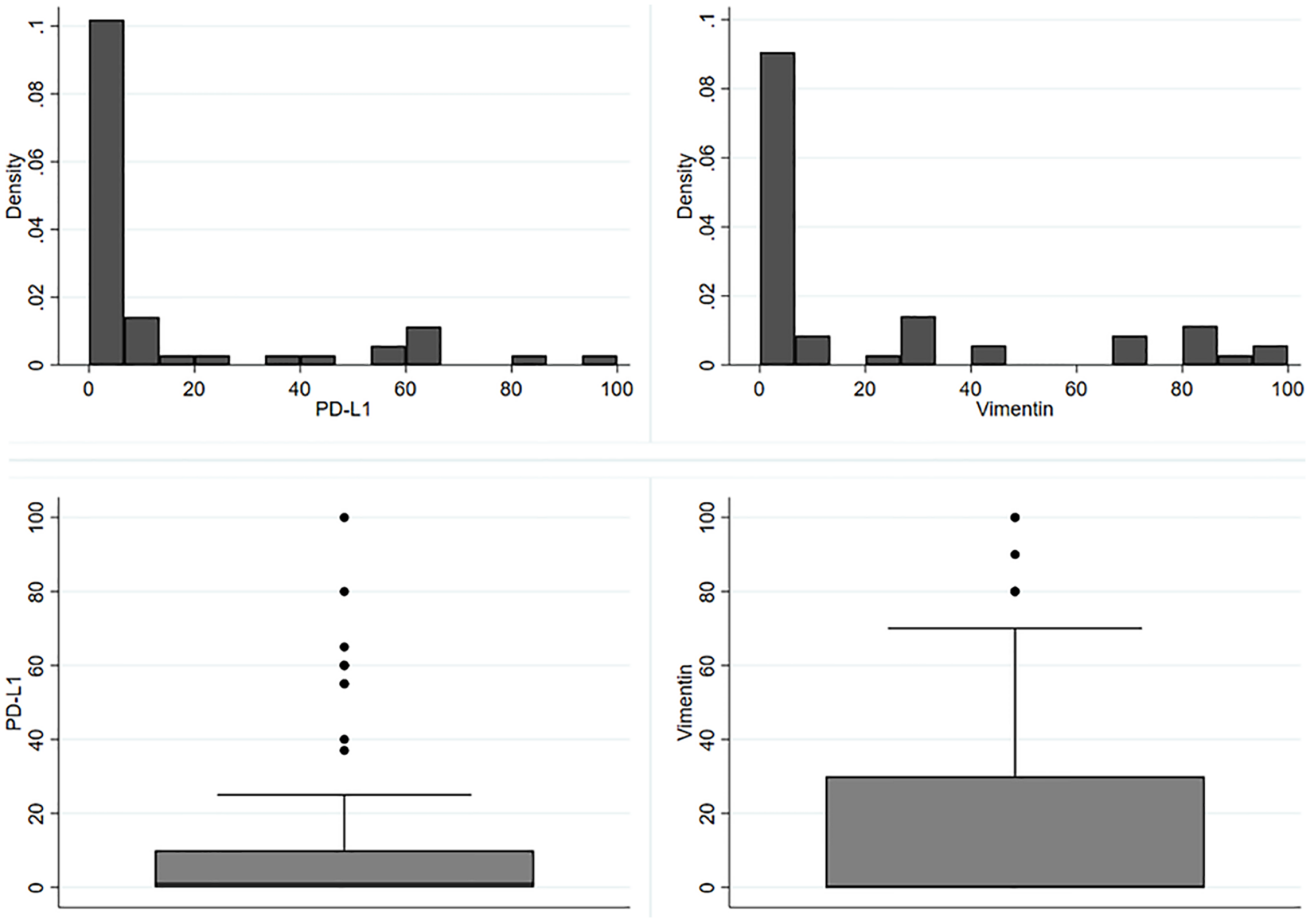
Expression value distribution for PD-L1 and vimentin.

**Figure 3:**
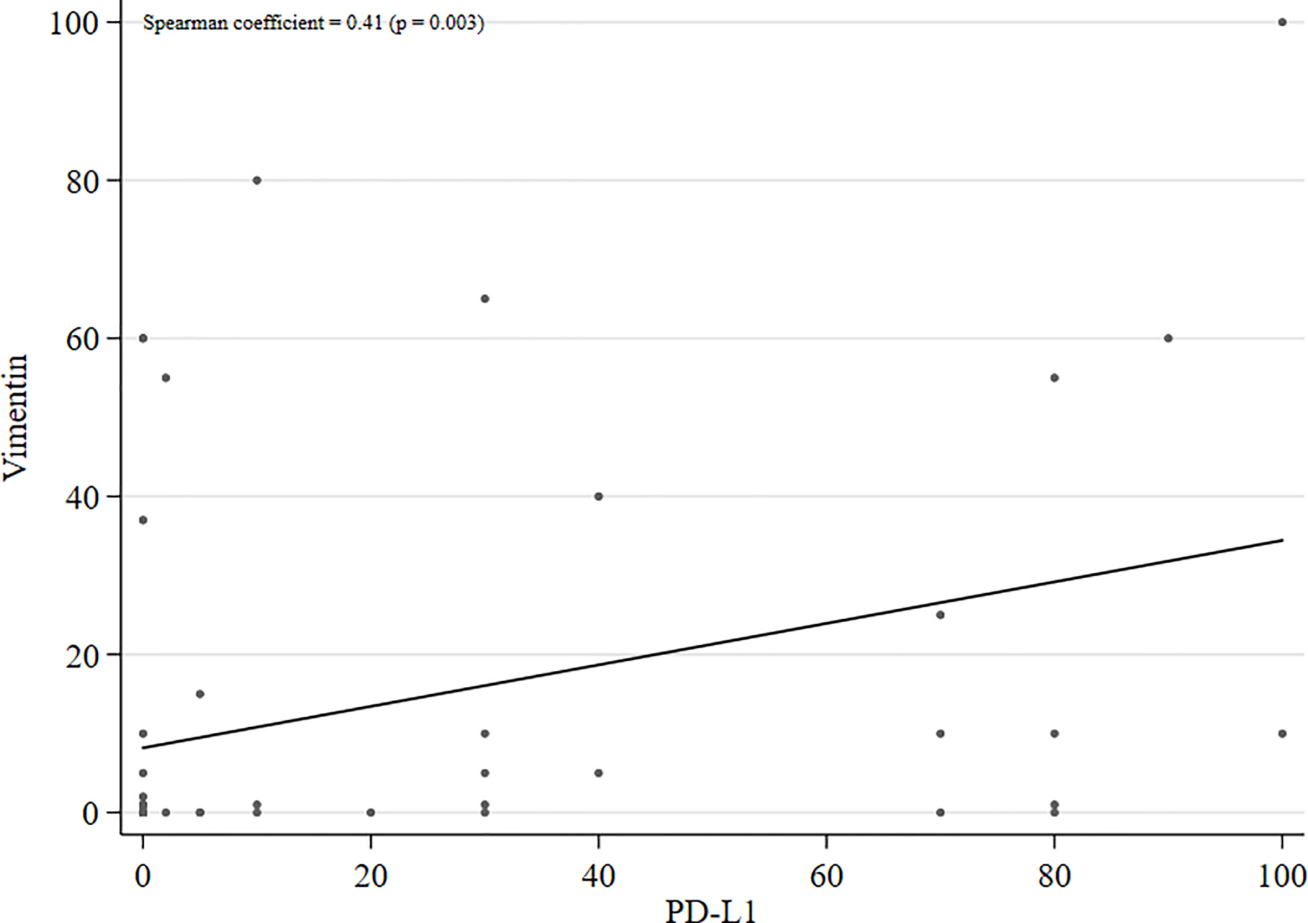
Scatter plot with regression line of PD-L1 and vimentin expression levels.

Fifty-three patients were included in the study. Patient characteristics are reported in **Table 1**. Mean age was 65 years (min-max: 47 - 87). The prevalent histology was non-squamous carcinoma (89%). Molecular characterization (KRAS, EGFR, ALK and ROS1 status) was not available for all of the patients. Twelve (23%) patients had previously undergone radical surgery. Only one line of systemic treatment for advanced disease was administered in 43% of patients, 23% underwent two lines of treatment and 34% 3 or more. **Figure 2** shows the distribution of PD-L1 and vimentin expression values. The median PD-L1 expression was 1 (min-max: 0 - 100), while that of vimentin was 0 (min-max: 0 - 100). Mean PD-L1 and vimentin expression was 14% (standard deviation: 25%) and 21% (standard deviation: 32%), respectively. Thirty (57%) patients were PD-L1-positive (≥1%) and twenty-six (49%) were vimentin-positive (≥1%). Both biomarkers were negative in 15 (28%) patients and positive in 18 (34%). In **Figure 3**, a scatter plot shows the relation between PD-L1 and vimentin expression values. The correlation, measured by the Spearman coefficient (*P* = 0.003), was 0.41, suggesting a weak positive correlation between the two biomarkers.

**Table 1:** Patient characteristics (*N* = 53).

Characteristics	*N (%)*
**Gender**	
Female	22 (41.51)
Male	31 (8.49)
**Age at diagnosis**	
Mean ± sd	65.45 ± 8.38
* Unknown*	2
**Stage at diagnosis**	
M0	14 (26.42)
M1	39 (73.58)
**Histology**	
Non-squamous	47 (88.68)
Squamous	6 (11.32)
**KRAS**	
Wild-type	24 (66.67)
Mutated	12 (33.33)
* Unknown*	17
**EGFR**	
Wild-type	41 (85.42)
Mutated	7 (14.58)
* Unknown*	5
**ALK**	
Negative	44 (97.78)
Positive	1 (2.22)
* Unknown*	8
**ROS1**	
Negative	44 (97.78)
Positive	1 (2.22)
* Unknown*	8
**Radical surgery**	
No	41 (77.36)
Yes	12 (22.64)
**Immunotherapy**	
No	48 (90.57)
Yes	5 (9.43)
**Chemotherapy**	
No	10 (18.87)
Yes	43 (81.13)
**Targeted Therapy**	
No	48 (90.57)
Yes	5 (9.43)

EGFR, epidermal growth factor receptor; ALK, anaplastic lymphoma kinase.

**Figure 4:**
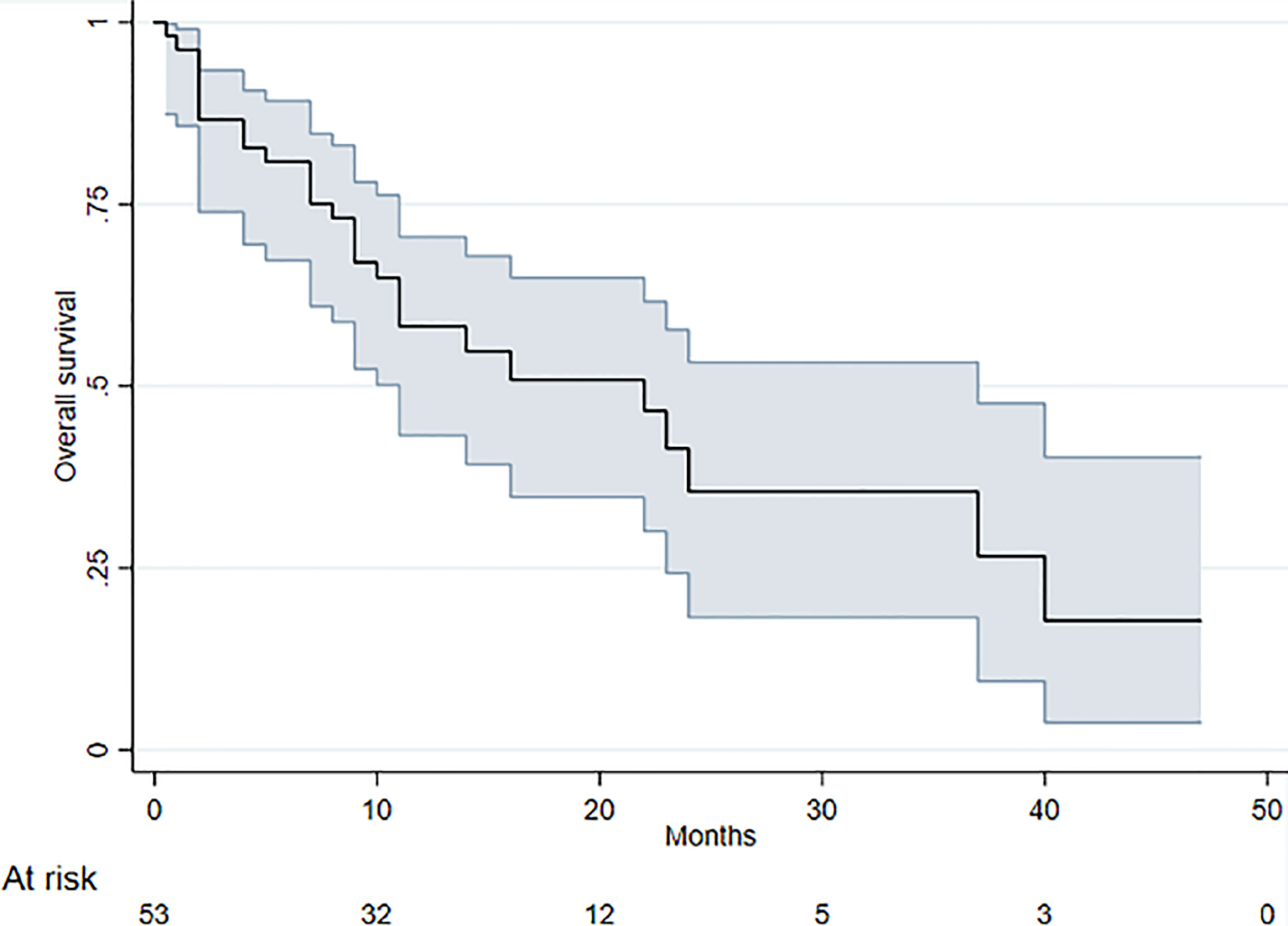
Kaplan-Meier curves for OS (shaded region corresponds to 95% confidence intervals).

**Figure 5:**
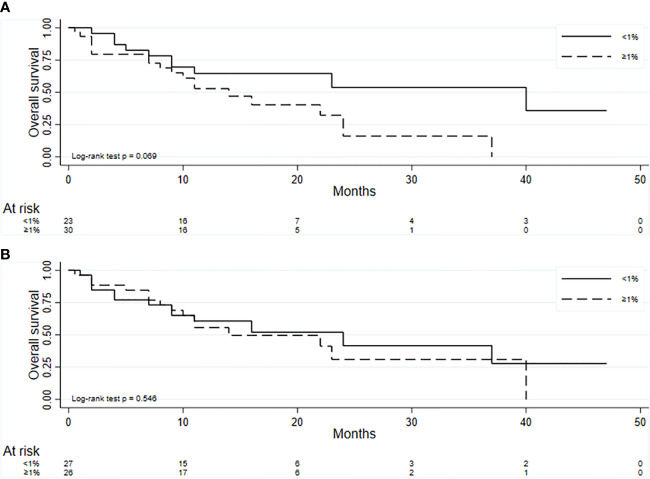
Kaplan-Meier curves for OS for PD-L1 **(A)** and vimentin **(B)**.

**Figure 6:**
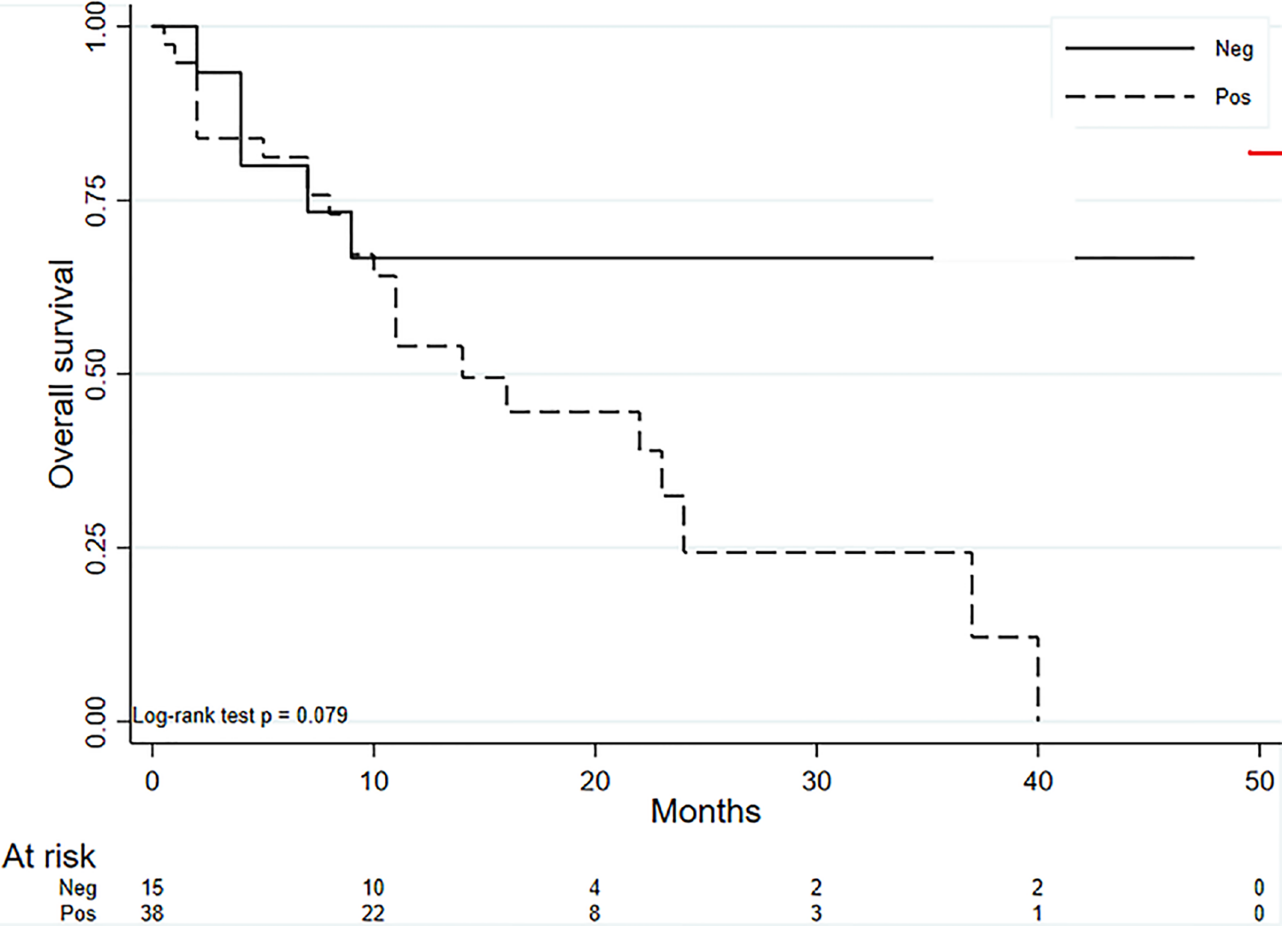
Kaplan-Meier curves for OS for negative PD-L1 and vimentin expression (<1%) and positive PD-L1 or vimentin expression (≥1%).

The median OS in our group of 53 patients was 22 months (95% CI 11-37), with 28 deaths recorded. **Figure 4** reports the Kaplan-Meier curves for OS. Patients with PD-L1 values <1% showed a slightly better OS than those with higher values (HR = 2.07; 95% CI: 0.92 – 4.65), but the difference was not significant (*P* = 0.080) (**Figure 5A**). No difference in survival was observed on the basis of vimentin expression (HR = 1.25; 95% CI: 0.59 – 2.66; *P* = 0.554) (**Figure 5B**). Patients with both vimentin and PD-L1 negativity (<1%) showed a better, albeit not significant, survival than those with PD-L1 or vimentin ≥1% (HR = 2.32; 95% CI: 0.87-6.17) (*P* = 0.093) (**Figure 6**). Univariate analyses for OS did not find significant results for gender, age at diagnosis, stage at diagnosis, histology, KRAS or EGFR status, radical surgery, and immunotherapy (**Table 2**).

**Table 2:** Results from Cox univariate analysis for OS.

	HR (95% CI)	*P*
**Gender**		
Female	1 (ref)	
Male	1.30 (0.61 – 2.78)	0.502
**Age at diagnosis, years**	0.99 (0.94 – 1.04)	0.750
**Stage at diagnosis**		
M0	1 (ref)	
M1	1.46 (0.52 – 3.73)	0.514
**Histology**		
Non-squamous	1 (ref)	
Squamous	2.12 (0.55 – 3.90)	0.449
**KRAS**		
Wild-type	1 (ref)	
Mutated	0.91 (0.35 – 2.38)	0.844
**EGFR**		
Wild-type	1 (ref)	
Mutated	0.87 (0.30 – 2.56)	0.804
**Radical surgery**		
No	1 (ref)	
Yes	0.47 (0.14 – 1.56)	0.217
**Immunotherapy**		
No	1 (ref)	
Yes	0.50 (0.22 – 1.10)	0.086
**PD-L1**		
<1%	1 (ref)	
≥1%	2.07 (0.92 – 4.65)	0.080
**Vimentin**		
<1%	1 (ref)	
≥1%	1.25 (0.59 – 2.66)	0.554

EGFR, epidermal growth factor receptor; PD-L1, programmed death ligand 1.

## Discussion

In our study we found the presence of a weak positive association between PD-L1 and vimentin. Moreover, the patients with both PD-L1 and vimentin <1% showed a better not significant survival than those with PD-L1 or vimentin ≥1%.

There is evidence of an interplay between EMT markers and immune checkpoint inhibitors (19–21). When EMT is activated in epithelial cells, PD-L1 is upregulated *via* the PI3K/AKT pathway and this process can be reversed by EMT suppression through microRNA-200 (22, 23). The association between PD-L1 expression and EMT phenotype in NSCLC has been already observed in tumor tissue and circulating tumor cells (24, 25), but some researchers have taken a more in-depth look at the relationship between the two markers. The work by Asgarova et al. showed that EMT induces immune evasion through the control of PD-L1 expression (26). Specifically, the authors found that the EMT phenotype was associated with PD-L1 upregulation through the synergistic exposure to TGF-β1 and TNF-α. They attributed this to the effect of TNF-α on NF-κB stimulation, which increases EMT induction *via* TGF-β1. NF-κB inhibition was also shown to block PD-L1 expression. These findings are corroborated by the fact that TGF-β1 and TNF-α induce global DNA demethylation, including the demethylation of the PD-L1 promoter, which determines higher PD-L1 expression.

The above findings attest to the effect of the EMT process on high PD-L1 expression. Interestingly, modulation of PD-L1 expression has been shown to influence EMT in esophageal cancer cells (27). In a recent work by Ancel et al., a threshold of ≥ 25% vimentin-positive tumor cells was significantly associated with poor tumor differentiation. However, vimentin expression alone was insufficient to predict a poor prognosis. The authors found that concurrent high PD-L1 and vimentin expression in early-stage NSCLC patients was more strongly associated with worse prognosis (28). Such findings are concordant with our finding of a trend towards a better prognosis in patients with low vimentin and PD-L1 expression. Furthermore, the slight association between PD-L1 and vimentin expression levels highlighted in our linear regression analysis is concordant with the known interplay between the EMT process and PD-L1 upregulation. This may be because other molecules may be involved in that interplay.

We are aware that our study has some limitations, *e.g.* its retrospective nature and the fact that it is based on a heterogeneous population of patients with advanced NSCLC who received various treatments. A prospective study of patients with a similar disease history would probably better address the issues raised. In addition, the number of patients included was small because of the frequent lack of archival tissue for vimentin determination. A prospective study design would, on the other hand, facilitate the collection of appropriate tissue samples. Moreover, the lack of a cut-off value to define vimentin and PD-L1 positivity limits a comparison with data from different studies.

Despite its limitations, our study suggests that EMT and immune escape may be components of the same process. It also indicates that research focused on new immune modulating agents and resistance mechanisms in NSCLC should take into account the role of EMT markers.

## Data Availability Statement

The original contributions presented in the study are included in the article/supplementary material. Further inquiries can be directed to the corresponding author.

## Ethics Statement

The studies involving human participants were reviewed and approved by Ethics Committee of IRST and Area Vasta Romagna. The patients/participants provided their written informed consent to participate in this study.

## Author Contributions

GB, FC, and LC conceived experiments; SB, MP and EP conceived experiments and analyzed data. SR, MMT and PU carried out experiments; AD, LL, PC, SS, and MB collected data. All authors contributed to the article and approved the submitted version.

## Conflict of Interest

The authors declare that the research was conducted in the absence of any commercial or financial relationships that could be construed as a potential conflict of interest.

## Abbreviations

NSCLC, non-small cell lung cancer; PD-1, programmed death 1; PD-L1, programmed death ligand 1; EMT, epithelial-to-mesenchymal transition; EGFR, epidermal growth factor receptor; TKI, tyrosine kinase inhibitor; TGF‐β, transforming growth factor beta; CSC, cancer stem cell; HR, hazard ratio; OS, overall survival; IHC, immunohistochemistry; SD, stable disease; CI, confidence interval; KRAS, Ki-ras2 Kirsten rat sarcoma viral oncogene homolog; ALK, anaplastic lymphoma kinase; ROS1, c-ros oncogene 1; TNF-α, tumour necrosis factor alpha; NF-κB, nuclear factor κB; PI3K, phosphatidylinositol-3’-kinase; AKT, protein kinase B.
